# Extracellular Vesicle-Mediated Metastasis Suppressors NME1 and NME2 Modify Lipid Metabolism in Fibroblasts

**DOI:** 10.3390/cancers14163913

**Published:** 2022-08-13

**Authors:** Barbara Mátyási, Gábor Petővári, Titanilla Dankó, Henriett Butz, István Likó, Péter Lőw, Isabelle Petit, Randa Bittar, Dominique Bonnefont-Rousselot, Zsolt Farkas, Tamás Szeniczey, Kinga Molnár, Krisztina Pálóczi, Edit I. Buzás, Mathieu Boissan, Anna Sebestyén, Krisztina Takács-Vellai

**Affiliations:** 1 Department of Biological Anthropology, Eötvös Loránd University, 1053 Budapest, Hungary; 2 Department of Pathology and Experimental Cancer Research, Semmelweis University, 1085 Budapest, Hungary; 3 Department of Molecular Genetics, National Institute of Oncology, 1122 Budapest, Hungary; 4 Hereditary Tumours Research Group, Hungarian Academy of Sciences, Semmelweis University, 1085 Budapest, Hungary; 5 Department of Anatomy, Cell and Developmental Biology, ELTE, Eötvös Loránd University, 1053 Budapest, Hungary; 6 Centre de Recherche Saint-Antoine, CRSA, Sorbonne Université, INSERM, 75012 Paris, France; 7 Service de Biochimie Métabolique, AP-HP, Sorbonne Université, Hôpitaux Universitaires Pitié-Salpêtrière-Charles Foix, 75013 Paris, France; 8 ICAN Maladies Cardiovasculaires et Métaboliques, Sorbonne Université, Inserm, UMR_S1166, 75013 Paris, France; 9 UFR de Pharmacie, Université Paris Cité, CNRS, Inserm, UTCBS, 75006 Paris, France; 10 Department of Genetics, Cell and Immunobiology, Semmelweis University, 1085 Budapest, Hungary; 11 HCEMM SU Extracellular Vesicles Research Group, 1503 Budapest, Hungary; 12 ELKH Translational Extracellular Vesicle Research Group, 1503 Budapest, Hungary

**Keywords:** NME, metastasis suppressor, extracellular vesicles, exosomes, lipid metabolism, fatty acid metabolism

## Abstract

**Simple Summary:**

Communication between cancer and stromal cells involves paracrine signalling mediated by extracellular vesicles (EVs). EVs transmit essential factors among cells of the tumour microenvironment. EVs derived from both cancer and stromal cells have been implicated in tumour progression. In this study, we focused on the first identified metastasis suppressor NME1, and on its close homolog NME2, and investigated their function in EVs in the interplay between cancer and stromal cells.

**Abstract:**

Nowadays, extracellular vesicles (EVs) raise a great interest as they are implicated in intercellular communication between cancer and stromal cells. Our aim was to understand how vesicular NME1 and NME2 released by breast cancer cells influence the tumour microenvironment. As a model, we used human invasive breast carcinoma cells overexpressing NME1 or NME2, and first analysed in detail the presence of both isoforms in EV subtypes by capillary Western immunoassay (WES) and immunoelectron microscopy. Data obtained by both methods showed that NME1 was present in medium-sized EVs or microvesicles, whereas NME2 was abundant in both microvesicles and small-sized EVs or exosomes. Next, human skin-derived fibroblasts were treated with NME1 or NME2 containing EVs, and subsequently mRNA expression changes in fibroblasts were examined. RNAseq results showed that the expression of fatty acid and cholesterol metabolism-related genes was decreased significantly in response to NME1 or NME2 containing EV treatment. We found that *FASN* (fatty acid synthase) and *ACSS2* (acyl-coenzyme A synthetase short-chain family member 2), related to fatty acid synthesis and oxidation, were underexpressed in NME1/2-EV-treated fibroblasts. Our data show an emerging link between NME-containing EVs and regulation of tumour metabolism.

## 1. Introduction

Metastases are responsible for 90% of death cases caused by cancer [1]. Therefore, understanding the biological functions of metastasis suppressor genes, which inhibit metastasis dissemination without influencing primary tumour growth, is essential [2]. The first metastasis suppressor gene identified was the mouse homologue of *NME1*, which was absent in metastatic, but present in non-metastatic mouse melanoma cell lines [3]. Since then, an additional nine members of the human NME protein family have been discovered. The most investigated members are NME1 and NME2, which are identical to 88% at the amino acid level and encode nucleoside diphosphate kinases (NDPKs) [4,5]. NME1 expression shows an inverse correlation with metastatic potential in melanoma, hepatocellular, breast and colon carcinoma, meaning that low NME1 level is connected to higher probability of metastasis formation in numerous tumour types [6,7,8,9,10,11,12,13,14,15]. However, in prostate and ovarian cancer, neuroblastoma or haematological malignancies, high NME1 expression is the hallmark of bad prognosis [16,17,18,19,20]. Our knowledge about the involvement of NME2 in different cancer types is rather limited: it has been implicated in oral squamous, breast, prostate, lung and ovarian cancers [11,21,22]. NME2 expression was significantly reduced in metastatic compared to non-metastatic tumour variants in some cohorts of patients with breast, lung and ovarian tumours [23]. Taken together, NME1 is the best studied metastasis suppressor, and the role of its highly homologous isoform, NME2, is much less documented in metastasis and remains controversial.

In addition, in neuroblastoma, breast carcinoma, acute myeloid leukaemia (AML) and non-Hodgkin lymphomas (NHL), peripheral T-cell lymphoma NME1 was detected in patients’ serum samples and its level showed a positive correlation with tumour progression [17,24,25,26,27]; thus, high serum NME1 level was associated with bad prognosis. Due to this correlation, many aspects of extracellular NME have been intensively investigated [28]. Recently, it was shown that in patients with breast carcinoma, NME1 is also secreted into the serum [27]. The secretome of colon cancer cell lines contained extracellular NME1 [29]. The presence of extracellular NME, more precisely NME2, was first detected in the supernatant of a mouse myeloid leukaemia cell line, where a subset of cells, also called M1 cells, were not able to differentiate into a more mature stage [30]. NME2 secretion was also detected from cells of human breast, colon, pancreas and lung tumours [31]. Both prokaryotic and eukaryotic NDPK/NME proteins can be secreted into the extracellular environment [32]. However, the way of secretion and the role of extracellular NDPKs (eNDPKs) are still little known. One of the possible roles of eNDPKs in the extracellular environment is the modulation of secreted nucleotide and nucleoside levels via their nucleoside diphosphate kinase activity. The nucleotides and nucleosides present in the extracellular environment act as signalling molecules through the purinergic signalling system, which is mediated through purinoceptors, divided into P1 adenosine receptors and P2 ATP and related nucleotides receptors [33,34,35]. The purinergic signalling system and extracellular NME proteins were linked in the cases of certain breast carcinoma cell lines [31]. Extracellular NME2 was suggested to play a role in modulation and regeneration of extracellular nucleotides, which can stimulate P2Y_1/2_ nucleotide receptors, which in turn take part in angiogenesis via activation of VEGFR-2 in the absence of its ligand VEGF [36,37].

As the NME1 isoform is present in patients’ serum samples and its level may correlate with stages of tumour progression in several tumour types, there is a need to understand the mechanism(s) by which NME is secreted into the extracellular environment. The tumour microenvironment represents a complex network, where intercellular communication among cancer cells and stromal cells is crucial and takes place by paracrine signals [38]. Accumulating evidence suggests that paracrine signalling can be mediated by EVs, which can transmit essential factors among cells in the tumour microenvironment [39].

In this study, we used human breast carcinoma cells and skin-derived fibroblasts as models to examine the role of EV-derived NME1/2 exerted on the microenvironment. Different EV fractions such as microvesicles or medium EVs (mEVs) and small EVs (sEVs), the best examples of which are exosomes, were isolated from the supernatant of NME1 and NME2 overexpressing breast carcinoma cells. We found that NME1 was present in microvesicles, whereas NME2 was abundant both in exosomal and microvesicular fractions. Next, we aimed to understand how extracellular vesicular NME1 and NME2 released by breast cancer cells influence the tumour microenvironment. Therefore, fibroblast cells representing a typical cell type of the tumour microenvironment were treated with NME1 and NME2 containing EVs, and subsequently mRNA expression changes of fibroblasts were examined. Our results show that the expression of fatty acid and cholesterol metabolism-related genes was decreased significantly in response to NME1 and NME2-EV treatment.

## 2. Results

### 2.1. NME1 and NME2 Are Present in EVs Derived from NME1/NME2 Overexpressing Human Breast Carcinoma Cell Lines

As a model, we used the invasive, human triple negative breast carcinoma cell line, MDA-MB-231T, which was stably transfected either by FLAG::NME1 or MYC::NME2 or the control vector [40]. Our aim was to examine the distribution of NME1 and NME2 in the EV fractions isolated from the supernatant of the above described cell lines. EVs of small size (sEV) containing exosomes and larger vesicles or microvesicles (mEV) [41] were isolated by differential centrifugation methods including ultracentrifugation from the culture media of the previously mentioned transfected MDA-MB-231T cells. The highly sensitive capillary Western immunoassay (WES^TM^) method was used to characterise lysates of the isolated EVs. Diameter distribution of EVs was controlled by nanoparticle tracking analysis (Figure S1).

**Figure 1:**
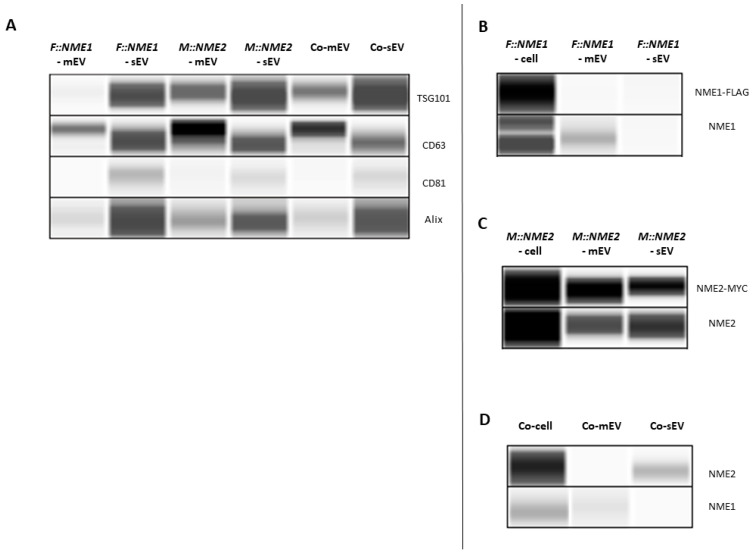
**Detection of NME1 and NME2 in extracellular vesicles (EVs) derived from supernatants of NME1 or NME2 overexpressing, and control vector expressing MDA-MB-231T cell lines with WES^TM^ Simple.** Abbreviations: F::NME1, M::NME2 and Co stand for protein or EV lysates from FLAG::NME1, MYC::NME2 and control vector-transfected cells, respectively. Small- and medium-size extracellular vesicles are abbreviated as sEV and mEV, respectively. (**A**) Vesicle marker detection in EV lysates. TSG101 content of sEV fraction was higher than that of the mEV fraction. The enrichment of CD63 was observed in both isolated sEV and mEV fractions. CD81 and Alix were predominantly present in sEVs. (**B**–**D**) Detection of NME1 and NME2 proteins in cell and EV lysates. (**B**,**D**) NME1 protein was present in cell extracts and mEV fractions of (**B**) FLAG::NME1, and (**D**) the control vector-transfected cells. (**B**,**D**) In sEVs NME1 was not detectable by either antibodies. (**C**,**D**) NME2 protein was detected in cell extracts and (**C**) in both mEV and sEV fractions of the MYC::NME2 expressing cell line by using anti-MYC and NME2 specific antibodies.

EV lysates were tested for presence of a series of different vesicle markers. CD81 and CD63 are plasma membrane-associated tetraspanins [42], while TSG101 and Alix are components of the endosomal sorting complex required for transport machinery (ESCRT) [43,44]. CD63 was enriched in both isolated exosomes and microvesicles (Figure 1A). CD81 surface marker and Alix cytosolic marker were present predominantly in exosomes (Figure 1A), whereas TSG101 was identified in a higher amount in exosomes but was also detectable in microvesicles (Figure 1A).

Next, we focused on the presence of NME homologs in EV lysates. We intended to detect the fusion proteins with antibodies specific for the tags. Although a higher amount of FLAG::NME1 derived from cell extracts was well visible on WES^TM^ expression profile (Figure 1B), in the case of EVs, the fusion protein was not detectable by the anti-FLAG antibody, presumably due to the low sensitivity of the antibody (Figure 1B). Therefore, subsequently we used a monoclonal antibody specific for NME1 (Figure 1B). NME1 protein was detected in cell extracts and the microvesicle fraction of FLAG::NME1-transfected cells (Figure 1B). Moreover, NME1 was also present in microvesicles of MDA-MB-231T cells transfected with the control vector (Figure 1D). In the sEV fraction, NME1 was not detectable by either antibody (Figure 1B,D).

MYC:NME2 content of EV lysates and cell extracts was examined using anti-MYC and selective NME2 specific antibodies [45]. The MYC tag detection worked efficiently both on cell extracts and EV lysates (Figure 1C). Anti-MYC and highly specific NME2 antibodies were able to recognise MYC::NME2 in both microvesicle and exosome fractions of the MYC::NME2 expressing cell line (Figure 1C). Endogenous NME2 was also detectable in exosomes of control vector-transfected cells, whereas endogenous NME1 was found in the microvesicles (Figure 1D).

**Figure 2:**
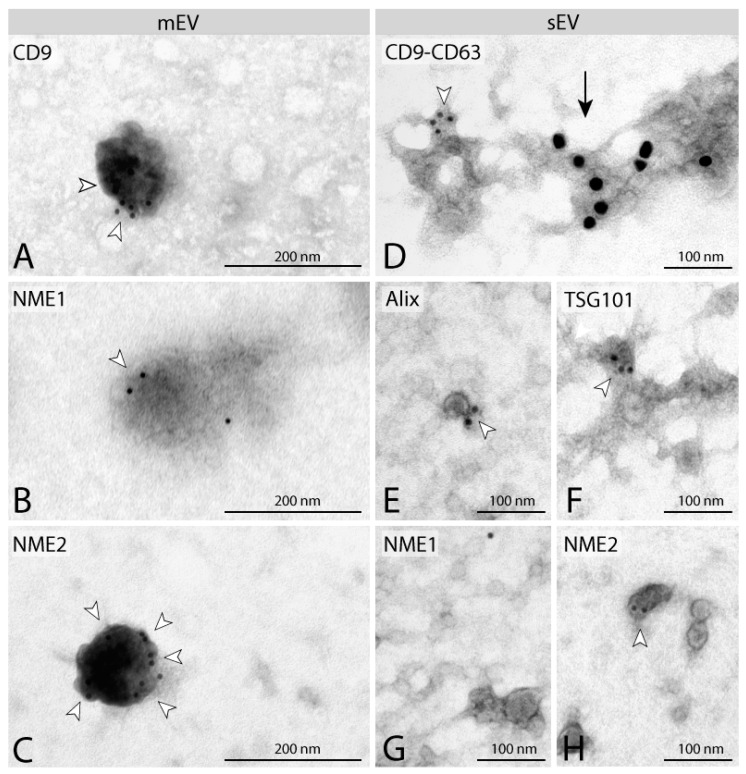
**Characterisation of separated EVs by immunoelectron microscopy.** Immuno-localisation of tetraspanins (CD9, CD63) on mEVs (**A**) and sEVs (**D**). EV markers Alix (**E**) and TSG101 (**F**) are present on sEVs. Identification of NME1 and NME2 on mEVs (**B**,**C**). NME1 cannot be detected on sEVs (**G**), whereas NME2 staining can be observed on sEVs (**H**). Gold particles are indicated by white arrowheads (10 nm) and black arrow (20 nm). mEVs and sEVs stained positive for EV markers CD63, CD9, Alix and TSG101 (**A**,**D**–**F**) were isolated from MDA-MB-231T cell line expressing the control vector pCDNA. NME1-positive mEVs (**B**) and NME1-negative sEVs (**G**) were isolated from F::NME1 overexpressing cells, whereas NME2 positive mEVs (**C**) and sEVs (**H**) are derived from M::NME2 overexpressing MDA-MB-231T cells.

In parallel with WES^TM^ measurements, immunoelectron microscopy was used to detect NME1 and NME2 in isolated microvesicle and exosome fractions. In this assay, Alix, TSG101, CD63 and another tetraspanin CD9 were used as positive EV markers. CD9 staining was observed on microvesicles (Figure 2A), whereas isolated sEVs stained positive for Alix and TSG101 (Figure 2E,F). In addition, isolated sEVs were labelled by anti-CD9-specific and anti-CD63-specific antibodies, using 10 and 20 nm diameter gold particles, respectively (Figure 2D). NME2 was detected on both isolated microvesicles (Figure 2C) and exosomes (Figure 2H), using an NME2 specific antibody, whereas NME1 was only found on isolated microvesicles (Figure 2B) but not on exosomes (Figure 2G), confirming the results obtained with WES.

### 2.2. Analysing the Transcriptomic Effect of NME1/NME2-Containing Microvesicles and Exosomes Exerted on Fibroblasts

Next, we intended to investigate the effect of NME1/2-containing EVs derived from breast carcinoma cells on fibroblasts, which are representative cells of the tumour microenvironment. Therefore, we treated patient-derived fibroblast cells (fibroblast 203-9) with either a mixture of both sEV and mEV derived from FLAG::NME1, MYC::NME2 or control vector-transfected MDA-MB-231T cells for 24 h, and performed a transcriptomic analysis of RNA extracted from these fibroblasts. In each treatment group, three biological replicates (three parallel fibroblast cultures treated with the appropriate EVs) were used.

The mRNA expression profile of fibroblast cells treated with EVs derived from NME1 or NME2 overexpressing MDA-MB-231T cells (NME1- or NME2-containing EVs) was compared to the fibroblasts, which received a treatment of EVs isolated from control vector-transfected breast carcinoma cells (control EVs: only two parallel samples were used).

**Figure 3:**
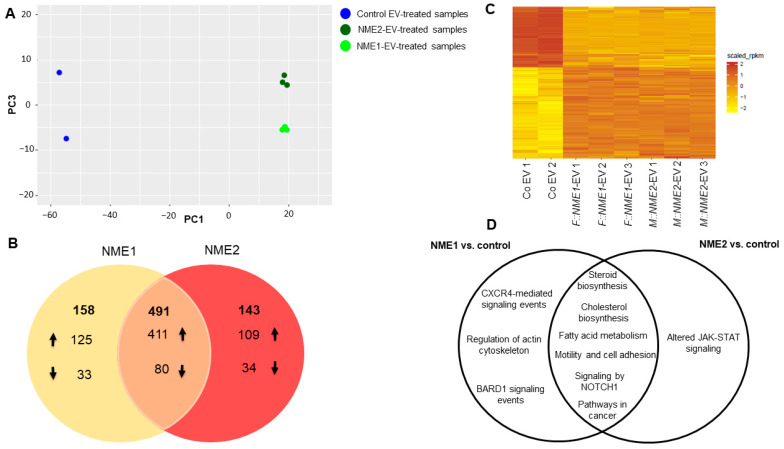
**Changes in transcriptomic signature of normal skin fibroblasts caused by NME1-EV and NME2 EV-treatments.** (**A**) Principal component analysis of 1370 differentially expressed CDS (FDR ≤ 0.01 AND logFC ≥ log2(2)). Control EV-treated samples are Co EV1 and Co EV2 (blue), NME1-EV treated fibroblasts are by represented by light green (F::NME1-EV1-3), whereas NME2- EV treated ones are labelled by dark green (M::NME2-EV1-3). (**B**) Venn diagram representing the summary of genes regulated by NME1- and NME2-containing EVs (FDR ≤ 0.05) in fibroblast cells. The number of up and downregulated genes is represented by up and down arrows; at the intersection, 491 genes can be found, which are up and downregulated by both NME1- and NME2-containing EVs. (**C**) Heatmap of RPKM values of 1370 differentially expressed CDS (FDR ≤ 0.01 AND logFC ≥ log2(2)). (**D**) Venn diagram of significant pathways, biological processes and molecular functions identified using KEGG, BioSystems: KEGG, MSigDB C2 BIOCARTA (v7.1) and PantherDB databases.

A principal component analysis based on the differentially expressed coding sequences (DE CDS) shows a distinct separation between control-EV and NME-EV-treated groups. PC3 axis also discriminates values of NME1-EV and NME2-EV-treated groups (Figure 3A). Here, 649 and 634 differentially expressed genes altered by NME1-EV and NME2-EV treatment were identified at a significant level (FDR ≤ 0.01 AND logFC ≥ log2(2)), respectively. Among NME1-EV regulated genes, 536 were upregulated and 113 downregulated. In the case of NME2-EV treatment, 520 genes were found to be upregulated, whereas 114 genes were downregulated. Overall, 491 common genes were identified, from which 411 are upregulated and 80 are downregulated by both NME1- and NME2-containing EVs (Figure 3B, Table S1). A heatmap of 1370 DE CDS also represents that a large group of genes are up- or downregulated by both NME1-EVs and NME2-EVs (Figure 3C). Pathway and gene ontology analysis identified alterations in steroid, cholesterol biosynthesis, fatty acid metabolism and NOTCH1 signalling in transcriptomic signature of fibroblasts treated either by NME1-EV or NME2-EV (Figure 3D). Perturbations in cancer-related pathways, such as motility and cell adhesion, were also detected in fibroblasts as a result of either NME1- or NME2-containing EVs. Unique changes followed by NME1-EV treatment were observed in CRCX4-mediated signalling events, regulation of actin cytoskeleton and BARD1-signalling events. NME2-EV treatment caused significant changes in JAK-STAT signalling (Figure 3D).

**Figure 4:**
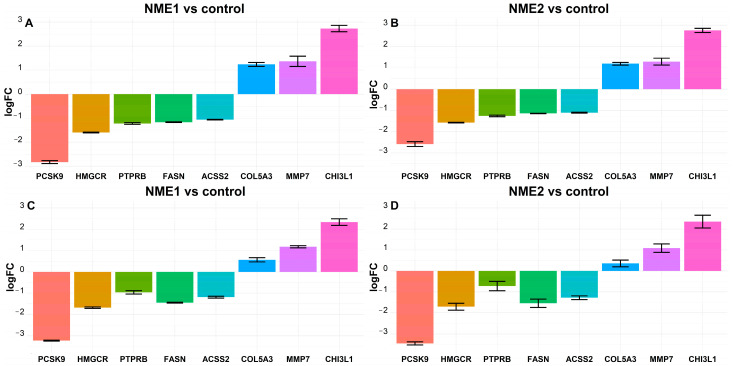
**Fatty acid and cholesterol metabolism-related genes are underexpressed, whereas some genes related to tumour progression are overexpressed in NME1-EV or NME2-EV treated fibroblasts.** Transcriptome analysis shows that the expression level of *ACSS2*, *FASN*, *HMGCR*, *PCSK9*, *PTPPRB* mRNAs was decreased, whereas the level of *CHIL3L1*, *COL5A3*, *MMP7* mRNAs was increased in fibroblasts in response to NME1-EV (**A**) and NME2-EV (**B**) treatment. LogFC values of the changes in gene expressions are shown relative to Co-EV treatment. (**C**,**D**) qPCR validation of the above-presented RNAseq data. qPCR results confirm expression changes obtained in transcriptome analysis. In the case of NME1-EV treatment, one-way ANOVA analysis revealed that there is a statistically significant difference in logFC values in both transciptomics and qPCR. The one-way ANOVA analysis of NME2-EV treatment yielded significant results for mean logFC differences in transcriptomics and qPCR data as well. The Tukey post-hoc test revealed that most of the pairwise comparisons are statistically significant.

Genes whose expression was decreased or increased to the largest extent in response to NME1- or NME2-EV treatment compared to the control condition were selected for further validation. Interestingly, a group of genes functioning in lipid and cholesterol metabolism showed the best match in this context: the expression of these genes showed a 3- to 8-fold decrease in fibroblast cells, when they received either NME1- or NME2-containing EVs. Among these genes, we found *PCSK9* (proprotein convertase subtilisin/kexin type 9) and *HMGCR* (3-hydroxy-3-methylglutaryl CoA reductase 2) playing a role in cholesterol metabolism. Lipid metabolism-related genes, *FASN* (fatty acid synthase) and *ACSS2* (acyl-coenzyme A synthetase short-chain family member 2), were also significantly underexpressed in NME-EV-treated fibroblasts (Figure 4A,B). Moreover, we found an angiogenesis-related gene *PTPRB* (protein tyrosine phosphatase receptor type B) also underexpressed (Figure 4A,B).

Among genes regulated by NME1-EVs or NME2-EVs in our dataset, which showed a significant change in expression and are known to be involved in processes of tumour progression, the following ones were selected for further analysis: *MMP7* (matrix-metalloproteinase 7) and *COL5A3* (collagen type V alpha 3 chain), which are related to metastasis/invasion, were significantly overexpressed; in addition, *CHI3L1* (chitinase 3-like 1) also showed an increase in expression in fibroblasts after NME1- or NME2-EV treatments (Figure 4A,B). These genes were subjected to real-time quantitative PCR (qPCR) to validate our data derived from transcriptome analysis. The expression of the following genes was significantly decreased in fibroblasts in response to treatment by NME1- or NME2-containing EVs compared to treatment managed by control EVs: *ACSS2*, *FASN*, *HMGCR*, *PCSK9*, *PTPRB* (Figure 4C,D). The expression level of *CHIL3L1*, *COL5A3* and *MMP7* genes was found to be increased when fibroblast cells received NME1- or NME2-containing EVs in comparison to control EVs (Figure 4C,D). Taken together, qPCR measurements nicely confirmed expression changes showed by RNAseq data (Figures S2–S5).

### 2.3. Fatty Acid Metabolism-Related Genes FASN and ACSS2 Are Downregulated in Fibroblasts Treated by NME1- and NME2-Containing EVs

Next, our aim was to examine whether expression changes resulted by NME1-EV or NME2-EV treatment can also be detected at the protein level. We selected two enzymes of fatty acid metabolism for further investigation, namely, ACSS2 and FASN, which are emerging markers of tumour metabolism. The function of fatty acid synthase (FASN) is crucial in growth and survival of tumours with lipogenic phenotypes [46]. Acyl coenzyme A synthetase 2 (ACSS2) converts acetate to acetyl-CoA and is linked to fatty acid oxidation [47]. Under hypoxic circumstances, ACSS2 can shift metabolism of cancer cells from aerobic glycolysis to oxidative phosphorylation (OXPHOS) [48]. NME1 and NME2 have not been linked to regulation of fatty acid synthesis and oxidation yet.

**Figure 5:**
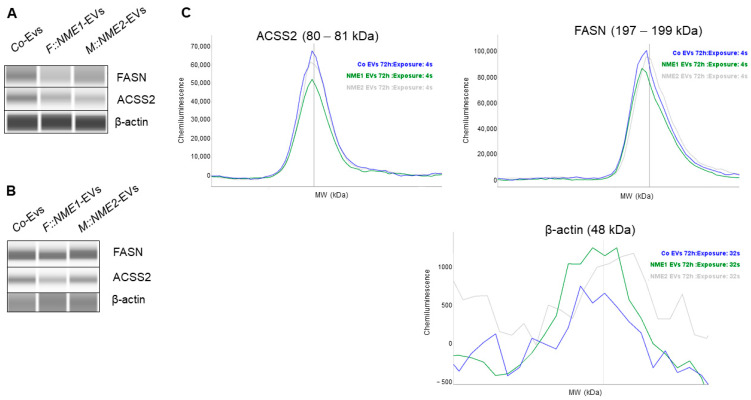
**FASN and ACSS2 protein levels are decreased in normal skin fibroblasts after NME1-EV and NME2-EV treatments**. Normal skin fibroblast cells were treated with a mixture of sEVs and mEVs derived from the supernatant of the three different transfected MDA-MB-231T cell lines: F::NME1, M::NME2 and Co, respectively. (**A**) After a 24 h incubation, fibroblasts were lysed, and protein extracts were prepared. Cell lysates were immunodetected by WES^TM^ Simple capillary immunoassay system. A decreased FASN and ACSS2 protein level was observed in fibroblasts treated with either NME1-EVs or NME2-EVs compared to the control condition. (**B**) In another experiment, fibroblasts were treated 3 times (0, 24, 48 h) with sEVs and mEVs derived from the supernatant of the three transfected MDA-MB-231T cell lines. After 72 h, cell lysates were collected and analysed by WES^TM^. (**C**) Corresponding electropherograms of WES^TM^ analysis on panel (**B**) also show that multiple NME1-EV and NME2-EV treatments resulted in decreased FASN and ACSS2 levels compared to the Co condition, analysed by WES^TM^ after 72 h.

To confirm reduced ACSS2 and FASN expression in response to NME1-EVs or NME2-EVs, we treated normal skin fibroblasts with a mixture of sEVs and mEVs from either NME1- or NME2-transfected cells for 24 h. Control treatment of fibroblasts was performed with a mixture of Co-EVs (microvesicles and exosomes). Each experiment was carried out in three biological replicates. Both proteins showed a decreased expression compared to the control condition when cells were treated with either NME1-EV or NME2-EV (Figure 5A). Next, NME1-EV and NME2-EV treatments were carried out three times, at different time points (0, 24 and 48 h), and FASN and ACSS2 levels of fibroblasts were examined after 72 h in protein extracts. Repeated EV treatment also resulted in decreased FASN and ACSS2 levels compared to the control condition (Figure 5B,C).

**Figure 6:**
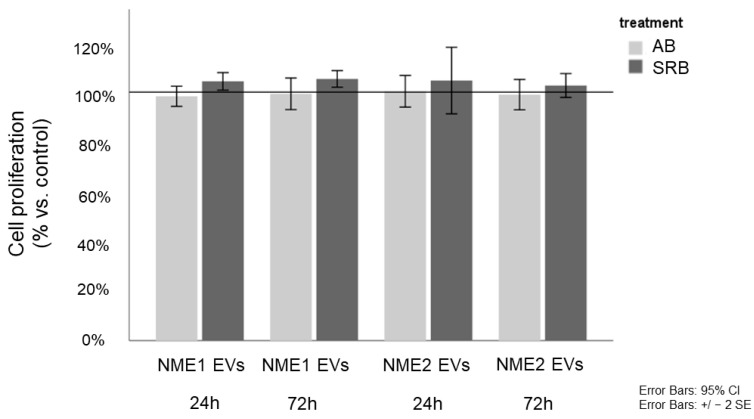
**NME1-EV or NME2-EV treatment does not influence proliferation of fibroblasts.** Alamar blue (AB) and sulforhodamine B (SRB) assays monitored the in vitro cell growth of EV-treated fibroblasts after 24 and 72 h. Cell proliferation of NME1-EV and NME2-EV treated fibroblasts is given in % of control (mean ± SD). Control corresponds to Co-EV treated fibroblasts. All data represent mean ± SD.

As we detected decreased FASN and ACSS2 levels in response to NME1-EV or NME2-EV treatment, we aimed to know whether the proliferation rate of fibroblasts was affected, assuming that low free fatty acid and cholesterol levels might negatively influence membrane synthesis. Proliferation assays of NME1-EV or NME2-EV treated and control-EV fibroblasts did not show any difference (Figure 6), indicating that fibroblasts treated with NME1-EV or NME2-EV did not acquire a property of reduced proliferation as compared to the Co-EV treatment.

### 2.4. GTEx Portal Data Show Low FASN, ACSS2, PCSK9 and HMGCR Levels but Relatively High NME1 and NME2 Expression in Normal Skin Fibroblasts

**Figure 7:**
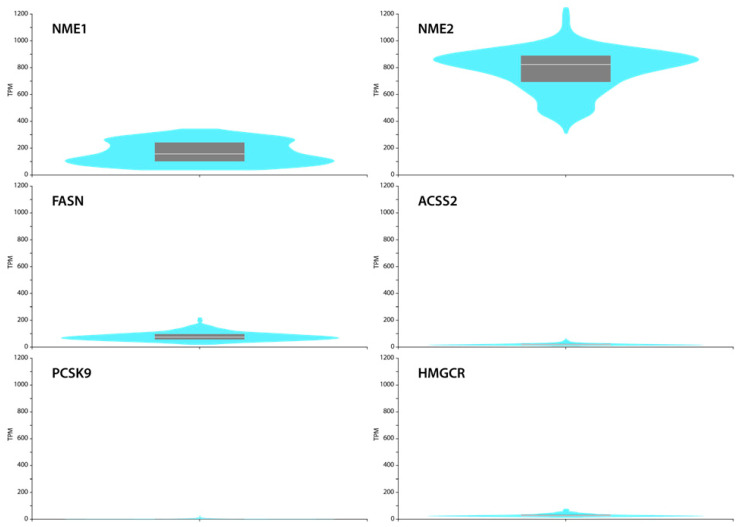
**GTEx Portal (a reference website) data show mRNA expression of NME1, NME2, FASN, ACSS2, PCSK9, HMGCR in cultured skin fibroblasts.** Box plots are shown as median and 25th and 75th percentiles; points are displayed as outliers if they are above or below 1.5 times the interquartile range. Expression values are shown in TPM (transcripts per million) in a linear representation.

To confirm these findings, we interrogated the GTEx Portal database (www.gtexportal.org, accessed on 1 July 2021) and analysed the mRNA expression data of NME1, NME2, FASN, ACSS2, PCSK9 and HMGCR in normal skin fibroblasts. Comparing the expression levels of those genes, we observed that NME1 and NME2 are expressed in fibroblasts, and in particular NME2, at a high level. The levels of NME1/2 target genes were low or hardly expressed. These observations suggest a negative correlation between NME expression and these target genes and confirm our transcriptional data (Figure 7).

## 3. Discussion

Extracellular NMEs have been shown to be present in sera of patients suffering numerous tumour types [17,24,25,26,27]. Moreover, high extracellular NME levels are often signs of bad prognosis and eNDPKs’ function was associated with tumour progression in the case of breast cancer [27]. Therefore, there is a need to understand how and why NME proteins can be released by the cancer cells into the extracellular environment. Both prokaryotic and eukaryotic NDPK/NME proteins can be secreted [32]. *Porphyromonas gingivalis*, an intracellular pathogen of the gingival epithelium, is able to translocate its bacterial NDPK into the extracellular environment using the pannexin-1-hemichannel (PNX1) of the host cell [49].

Nowadays, EVs raise a great interest because most EVs are tools of intercellular communication as they carry specific proteins, lipids and nucleic acids, and thus EVs are emerging targets of both tumour diagnosis and therapy. Among EVs, mainly exosomes are implicated in crosstalk of cancer and microenvironmental cells [39]. In addition, new technologies are emerging based on association of specific markers with exosome subpopulations, which allow engineering of target-guided exosome-like particles for potential use in clinical practice [50]. Therefore, identification of specific markers associated with different exosome populations is of key importance. Application of exosome-based nanoplatforms have been suggested in breast cancer therapy as well [51].

In this study, we used NME1 and NME2 overexpressing triple negative breast carcinoma cells as a model, and first examined the presence of the NMEs in microvesicular (mEV) and exosomal (sEV) fractions derived from their supernatants. NME2 is present in higher quantities in EVs, whereas NME1 is detected in a lower level. We first performed a detailed subtype-specific analysis of breast carcinoma cell-derived EVs, which showed that NME1 was only present in mEVs. In contrast, NME2 was abundant in both mEVs and sEVs. In addition, we also detected the endogenous NME isoforms in the corresponding sEV and mEV fractions of control vector transfected cells.

Next, we treated normal skin fibroblasts by a mixture of microvesicles and small EVs from NME1 or NME2 overexpressing breast carcinoma cells and then examined their effect on the RNA pool of fibroblasts. Pathway analysis data show that expression of genes involved in lipid metabolism such as cholesterol and fatty acid synthesis strongly decreased in response to NME1 or NME2 EV treatment. We focused on this group of genes as recently a novel link has emerged between lipid metabolism-related genes and NMEs [52]. We note, as a limitation of our study, that the data are obtained from human skin-derived fibroblasts of a single patient.

PCSK9 and HMGCR contribute to cholesterol biosynthesis, whereas FASN and ACSS2 are involved in fatty acid synthesis and oxidation, respectively. Our data show that as a result of NME1-EV or NME2-EV treatment, *PCSK9, HMGCR, FASN* and *ACSS2* mRNA levels decreased to 3- to 8-fold in normal skin fibroblasts, compared to the treatment managed by control vector-containing EVs. Preliminary data show that free fatty acid levels decreased in NME1-EV-treated fibroblasts, which might be the consequence of reduced FASN levels; however, this issue needs further investigation. We also analysed in detail the expression pattern of normal skin fibroblasts: GTEx Portal data show that high expression of NME2 and moderate expression of NME1 is possibly linked to low PCSK9, HMGCR, FASN and ACSS2 levels. Based on these, we suggest that high levels of NME1 and NME2 through EV might be responsible for maintaining PCSK9, HMGCR, FASN and ACSS2 expressions at low levels in fibroblastic stromal cells.

Fibroblasts are key components of the tumour microenvironment. A parallel dialogue exists between cancer cells and fibroblasts, which first helps cancer cells to attract normal fibroblasts, and next transforms disease-free fibroblasts into cancer-associated fibroblasts (CAFs). CAFs, in turn, can support and modulate cancer cells (reviewed in [53]). Due to this crosstalk, which can be managed by direct contact through cytokines or EVs, CAFs modulate the extracellular matrix, promote invasion or even facilitate EMT [53].

Cancer cells were also shown to induce metabolic changes in stromal cells; these changes allow CAFs to display activities, which support growth and progression of cancer cells. Metabolic adaptation is recognised as a hallmark of cancer [38]: crosstalk of cancer cells and cells forming the tumour microenvironment results in a metabolic symbiosis during tumour progression [54]. For example, in an orthotopic mouse model for ovarian carcinoma, glutamine supply of cancer cells was ensured by cancer-associated fibroblasts [55]. In colorectal cancer, cancer-associated fibroblasts underwent lipidomic reprogramming (among others, FASN overexpression) and secreted lipid metabolites for cancer cells; in this study, the uptake of secreted lipids was followed by enhanced migration of colorectal cancer cells [56].

Emerging data suggest a close connection between altered lipid metabolism and tumour progression, and most evidence shows that higher lipid levels, which are critical for membrane production and energy generation, support tumour progression [57]. Several tumour types, such as melanoma [58] or pancreatic cancer [59], can be characterised by lipid abundance. In addition, recent data show that lipids negatively influence antitumour immune response [60].

Studies conducted on invasive triple negative breast cancer cells and cancer-associated fibroblasts also identified fibroblasts as hubs and cancer cells as gatherers of lipids [61]. MDA-MB-231 cells, whose FASN levels are low [62], can cover their lipid demand by using exogenous sources provided by cancer-associated fibroblasts in the microenvironment [61,63]. MDA-MB-231 cells exposed to fibroblast-conditioned media displayed decreased lipid chaperon (FABP) but increased lipid transporter (FATP) expression, signs of lipid transfer from cancer-associated fibroblasts to cancer cells [61].

Compared to the above data, in our study we modelled an early stage of metabolic symbiosis between triple negative breast carcinoma cells and normal fibroblasts, when fibroblasts are still not transformed to cancer-associated fibroblasts. In this setup, we showed that MDA-MB-231T-derived EV-mediated NME1 and NME2 induce decreased fatty acid synthesis with low FASN and ACSS2 levels in normal fibroblasts, without influencing proliferation of fibroblasts. We suggest that EV-derived NME1 and NME2 cause decreased lipid production in fibroblasts, thereby inhibiting lipid supply of cancer cells provided by fibroblasts at this early stage of metabolic symbiosis.

We also hypothesise that decreased lipid production induces metabolic changes in fibroblasts, which might result in epigenetic reprogramming. To further investigate this question, metabolomic and lipidomic analyses of fibroblasts could be performed.

## 4. Conclusions

This study established that: (1) EVs of NME1 overexpressing breast carcinoma cells contained NME1 in mEVs but not in sEVs. (2) NME2 is present in both mEV and sEV fractions of NME2 overexpressing breast carcinoma cells. (3) NME2 is more abundant in EVs compared to NME1. (4) NME1-EV and NME2-EV treatment of normal skin fibroblasts results in underexpression of fatty acid and cholesterol metabolism genes. (5) Decreased FASN and ACSS2 protein levels were detected in fibroblasts followed by NME1-EV or NME2-EV treatment, which was not accompanied by a change in their proliferation.

Our data show an emerging link between extracellular vesicular NMEs and markers of tumour metabolism. EV-mediated NME1 and NME2 might inhibit supporting activities of fibroblasts by inducing decreased fatty acid and cholesterol synthesis.

## 5. Materials and Methods

### 5.1. Cell Lines

To examine the presence of NME1 and NME2 in EVs, we used human breast adenocarcinoma MDA-MB-231T cell lines, which were stably transfected with one of the following constructs: pcDNA3 (Co), pcDNA3/FLAG-NME1 (F::NME1) or pcDNA3/MYC-NME2 (M::NME2) (the clones are a kind donation of Maja Herak Bosnar (Rudjer Boskovic Institute, Zagreb, Croatia)). These cell lines were cultured in DMEM high glucose media (Biosera, Nuaille, France) supplemented with 10% FBS (Biosera), 2 mM L-glutamine (Biosera), 100 µI/mL penicillin–streptomycin (Biosera), 7.5% NaHCO3 (Biosera) and 50 mg/mL geneticin (Sigma) at 37 °C with 5% CO_2_.

To monitor the effect of EVs derived from MDA-MB-231T cells, we applied a human skin derived cell line, fibroblast 203-9. They were cultured in DMEM high glucose media (Biosera) supplemented with 10% FBS (Biosera), 2 mM L-glutamine (Biosera), 100 µI/mL penicillin–streptomycin (Biosera), 1% sodium pyruvate (Biosera) at 37 °C with 5% CO_2_.

### 5.2. Extracellular Vesicle Isolation from Conditioned Cell Culture Medium

During extracellular vesicle purification, two size-based subpopulations (mEVs and sEVs) were isolated by differential centrifugation/ultracentrifugation techniques. Twenty-four hours before EV isolation, cells were washed with PBS and maintained at the same conditions as we described above, with one exception: the replaced culture media contained 10% EV-free FBS (ultracentrifuged at 120,000× *g* for 15 h). After 24 h, the conditioned media were collected and centrifuged 2 times at 200× *g* for 5 min at room temperature to remove cells. Then the supernatant was centrifuged at 2000× *g* for 20 min at 16 °C (Avanti J-XP26 centrifuge, JS 5.3 rotor, Beckman Coulter Inc., Pasadena, CA, USA) and filtered by gravity through 5 μm (Milipore) and 0.8 μm (Whatman) filters. Next, supernatant was centrifuged at 12,500× *g* for 20 min at 14 °C to pellet mEVs (Avanti J-XP26 centrifuge, JA 25.15 rotor, Beckman Coulter Inc.). Subsequently, supernatant was filtered again by gravity through a 0.22 μm (Filtropour, Sarstedt, Germany) membrane and centrifuged at 100,000× *g* for 70 min at 4 °C using an MLA-55 rotor in an Optima Max XP ultracentrifuge to pellet sEVs (Beckman-Coulter). Both mEVs and sEVs pellets were resuspended once in NaCl-HEPES (0.9% NaCl containing 10 mM HEPES, pH 7.4), and recentrifuged under the same conditions as used originally for pelleting.

### 5.3. Nanoparticle Tracking Analysis (NTA) of EVs

The size distribution and concentration of EVs were analysed by NTA on a ZetaView PMX-120 instrument (Particle Metrix, Inning am Ammersee, Germany). All samples were diluted in NaCl-HEPES to a final volume of 1 mL. The manufacturer’s default software settings were selected for EV analysis. For each measurement, two cycles were performed by scanning 11 cell positions. The following camera settings were used: Shutter: 100 (sEVs), 150 (mEVs); Sensitivity: 85 (sEVs), 75 (mEVs); Framrate: 60 (sEVs), 7.5 (mEVs); cell temperature: 25 °C. The videos were analysed with minimum brightness of 20, a minimum area of 5 and a maximum area of 1000 by ZetaView Analyze software 8.05.10.

### 5.4. Detection of EVs Content by Immunoelectron Microscopy

In this study, 5 µL of separated vesicle sample aliquots were dropped onto formvar/carbon coated 200-mesh nickel grids (S160N3, AGAR Scientific Ltd., Essex, UK). After 40 min of drying, grids were floated upside down on 50 μL drops of the following solutions: blocking solution (3% non-fat milk powder in TBS, T-8793, Sigma, St. Louis, USA), one of the primary antibody solutions (anti-Alix 1:100, anti-CD9 1:50, anti-CD63 1:50, anti-NME-1 1:100, anti-NME-2 1:100, anti-TSG101 1:100) and the secondary antibody solution (10 nm gold conjugated anti-rabbit 1:50, G3779-.4ML, Sigma, USA; 10 nm gold conjugated anti-mouse 1:50, G7652-. 4ML Sigma, USA; 20 nm gold conjugated anti-mouse, 1:50, ab27242, Abcam, UK). Antibodies are described in the table below. Incubation times were 30 min, 60 min and 4 h, respectively. All antibodies were diluted in 1.5% non-fat milk powder/TBS. TBS-1% BSA washing steps were inserted between incubations. Finally, grids were contrasted with 2% uranyl acetate prior to the investigation by a JEOL JEM-1011 electron microscope equipped with a high resolution Morada CCD camera from Olympus Soft-Imaging-Solutions.

### 5.5. EV Treatment of Fibroblasts and RNA Isolation

Fibroblast 203-9 cells were treated with EVs derived from MDA-MB-231T cells. Fibroblasts were cultured in 6-well plates at the same conditions mentioned above. Before treatment, the wells were gently washed two times with PBS and then the mixture of the isolated sEVs and mEVs was added and incubated for 24 h at 37 °C with 5% CO_2_. We tested three different groups of samples: three parallel fibroblasts cultures were treated with either FLAG::NME1 or MYC::NME2-containing vesicles; control samples received EVs isolated from MDA-MB-231T cells overexpressing the control vector pCDNA (Co-EV treatment). Each experiment was carried out in 3 biological replicates, except for Co-EV -treated samples, where 2 independent samples were used. After a 24 h incubation, fibroblasts were lysed, and RNA isolation was performed with RNeasy Mini kit (Qiagen, Hilden, Germany). The RNA concentration and purity were measured by NanoDrop (ND-100, RNA-40).

### 5.6. Transcriptome Analysis

#### 5.6.1. Transcriptome Sequencing

NEBNext Ultra II Directional RNA Library Prep Kit for Illumina with Purification Beads (NEB #E7760S/L) was used for PolyA NGS library preparation following the manufacturer’s instructions. Sequencing was run on Illumina NovaSeq platform (NovaSeq 6000 SP 300 cycles (2 × 150 bp)) with data output 100 M PE reads/sample.

#### 5.6.2. Genome Sequence and Gene Annotations

The hg19 assemblies were obtained from Ensembl database. The chromosomal sequences and annotation files were downloaded as fasta and gtf formats, respectively. Genome and transcriptome sequences in fasta format were indexed with Tophat2 (Bowtie2).

#### 5.6.3. Pre-Processing of RNAseq Data

Illumina paired end sequencing data were exported in FASTQ file format. The reads were trimmed using Trim Galore (Babraham Bioinformatics, https://www.bioinformatics.babraham.ac.uk/projects/trim_galore/, accessed on 19 November 2019) and cutadapt [64] to remove bases where the PHRED quality value was less than 20. Potential 3’ adapter and poly(A)-tail fragments were also removed. 

#### 5.6.4. Alignment of Obtained Reads to Genomes

The trimmed sequence sets were aligned to the genome with Tophat2 (Bowtie2), with default parameters. For further analysis the reads were sorted by samtools (http://samtools.sourceforge.net, accessed on 19 November 2019) according to coordinates [64,65,66].

The function featureCounts (Rsubread R package) was used for counting reads to genomic features. Count-based gene expression estimation with union-CDS based counting were used. To implement union-CDS, we selected the CDS entries from the annotation files, grouped them by the ENSEMBL gene identifier and merged overlapping CDS for each gene [67].

#### 5.6.5. Testing for DE Genes

The Bioconductor package edgeR was used for differential expression analyses. Features with very low counts across all libraries were filtered out prior to further analysis.

The GLM likelihood ratio test was used with the Cox–Reid dispersion estimates. Multiplicity correction was performed by applying the Benjamini–Hochberg method on the *p*-values, to control the false discovery rate (FDR) [68,69].

A heatmap was generated based on RPKM values of 1370 differentially expressed CDS (FDR ≤ 0.01 AND logFC ≥ log2(2)) (Figure 3C).

#### 5.6.6. Bioinformatic Analysis

Functional (pathway and gene ontology) analyses were performed using ToppGene Suite [70].

Gene set enrichment analyses were performed for Gene Ontology categories (Biological Processes, Molecular Function and Cell Component) and KEGG, BioSystems: KEGG, MSigDB C2 BIOCARTA (v7.1) and PantherDB Pathways gene sets. Results were considered to be significant with *p* < 0.05. We used the Benjamini–Hochberg method to control the false discovery rate at 0.05.

### 5.7. qPCR Experiments

For quantitative real-time PCR (qRT-PCR) experiments, 350 ng of total RNA was reverse transcribed using High-Capacity RNA-to-cDNA Kit (#4368814, Thermo Fisher Scientific, Waltham, MA, USA) according to the manufacturer’s instructions. Predesigned TaqMan Gene Expression assays were used for gene expression measurements (Human FASN: Hs01005622_m1, ACCS2: Hs00218766_m1, PCSK9: Hs03037355_m1, CHI3L1: Hs01072230_g1, PTPRB: Hs01549032_m1, MMP7: Hs01042796_m1, HMGCR: Hs00168352_m1, COL5A3: Hs01555669_m1, ACTB: Hs01060665_g1 all from Applied Biosystems by Life Technologies). cDNA was diluted 100x. All measurements were performed in triplicate. DeltaCT (dCT) values were calculated and deltadeltaCT (ddCT) values were normalised to the controls in the experiments. Fold change values were calculated from 2−ddCT.

### 5.8. EV Treatment of Fibroblasts and Protein Isolation

Fibroblast 203-9 cells were treated with EVs derived from MDA-MB-231T cells. Fibroblasts were cultured in 6-well plates and treated by a mixture of NME1-EVs, NME2-EVs and Co-EVs as mentioned above (Section 5.6). Again, we tested three different groups of samples: three parallel fibroblast cultures were treated with either FLAG::NME1 or MYC::NME2-containing vesicles; control samples received EVs isolated from MDA-MB-231T cells overexpressing the control vector pCDNA (Co-EV treatment). After a 24 h incubation, fibroblasts were lysed, and protein isolation was performed. Protein lysates were further examined by WES (Section 5.9).

### 5.9. Capillary Western Immunoassay (WES^TM^)

The protein content of cell lysates and EV lysates was quantified by Bradford protein assay (BioRad, Hercules, CA, USA) and immunodetected with WES^TM^ Simple analysis on WES^TM^ system (ProteinSimple-Biotechne 004–600). Here, 12–230 kDa Separation Module (ProteinSimple SM-W004) and either the Anti-Mouse Detection Module (ProteinSimple DM-002) or Anti-Rabbit Detection Module (ProteinSimple DM-001) were applied depending on the primary antibodies. Briefly, protein samples were diluted in sample buffer thereafter mixed with Fluorescent Master Mix 1:4 and denatured at 95 °C for 5 min. According to the manufacturer’s instructions, the samples, the blocking reagent (antibody diluent), the primary antibodies, the HRP-conjugated secondary antibodies and the chemiluminescent substrate were added to the plate. Immunodetection was performed automatically, and the results were reported as virtual gels and electropherograms. The default settings were the following: stacking and separation at 395 V for 30 min; blocking reagent for 5 min, primary and secondary antibodies both for 30 min; luminol/peroxide chemiluminescence detection for 15 min (exposure times were selected for different antibodies between 1 and 512 s). Primary antibodies used are detailed in the Table 1: 

### 5.10. In Vitro Proliferation Assays—Alamar Blue and Sulforhodamine B Assays

Alamar blue (AB; 10 µL/well; Thermo Fisher Scientific) assay was applied to monitor the short-time effect (24 and 72 h) of EVs on fibroblast cell growth and cellular metabolic activity. After a 4 h incubation with AB, the fluorescence was measured with a fluorimeter (Fluoroskan Ascent FL; Labsystems International; Ascent Software, Fluoroskan Ascent, Vantaa, Finland) at 570–590 nm. To detect the total protein content of control and EV-treated cells, sulforhodamine B (SRB) test was performed. Fibroblasts were fixed by adding trichloroacetic acid (10%; 50 µL/well) for one hour at 4 °C; then, the wells were washed with distilled water. After a 15 min incubation with SRB (0.4 m/V%; 50 µL/well) at room temperature, the unbound SRB solution was washed with acetic acid (1%). Thereafter, Tris base solution (10 mM; 150 µL/well) was added to each well to solubilise the protein-bound dye. Multiskan MS microplate reader (Labsystems International; Transmit Software, Vantaa, Finland) was used for measuring the absorbance at 570 nm.

### 5.11. Free Fatty Acid Measurements

Free fatty acid levels were determined by enzymatic colorimetric method (DiaSys) on a Konelab 20i Analyzer (Thermo Fischer Scientific, Waltham, MA, USA).

### 5.12. Statistical Analysis

In case of transcriptomics and qPCR data, the differences in the logFC values of the investigated genes were examined using one-way analysis of variance (ANOVA) with Tukey’s post-hoc test for multiple comparisons. The proliferation assay was evaluated with an independent samples *t*-test. The WES^TM^ data were analysed with a one-sample *t*-test. In every analysis, *p*-values less than 0.05 were considered statistically significant.

## Figures and Tables

**Table 1:** List of the used primary antibodies (catalogue numbers, dilutions and expected sizes for WES analyses were given).

Primary Antibody	WES		Electron Microscopy		Size (kDa)
	Cat. No.	Dilution	Cat. No.	Dilution	
NME1	*OriGene* #TA801264	1:50	*OriGene* #TA801264	1:100	17
NME2	[45]	1:50	[45]	1:100	17
FLAG (biotin)	*Sigma* #F9291	1:50	-	-	17
MYC	*Sigma* #M4439	1:50	-	-	17, 62
β-Actin	*Sigma* #A2228	1:100	-	-	42
CD63	*Santa Cruz* #sc-15363	1:50	*Abcam* #ab134045	1:50	26/30, 60
CD81	*Sigma* #SAB3500454	1:50	-	-	26
CD9	-	-	*Sigma* #C9993	1:50	24
Alix	*Sigma* #SAB4200477	1:50	*Abcam* #ab186429	1:100	95
TSG101	*Sigma* #T5701	1:50	*Sigma* #T5701	1:100	46
FASN	*Cell signaling* CS #3180	1:50	-	-	241
ACSS2	*Cell signaling* CS #3658	1:50	-	-	79

## Data Availability

GEO number can be provided up on request from correspondence author.

## Supplementary Materials

The following supporting information can be downloaded at: https://www.mdpi.com/article/10.3390/cancers14163913/s1, Figure S1: Nanoparticle tracking analysis (NTA) of Evs. Size distribution and concentration of (A, C) sEVs and (B, C) mEVs. Table S1: Common genes regulated by NME1-EVs and NME2-EVs in fibroblasts. Figure S2: One-way ANOVA analysis of genes selected from transcriptome analysis (see Figure 4B: NME2-EV vs. Co-EV treatment) reveals that most of the pairwise comparisons are statistically significant. Figure S3: One-way ANOVA analysis of genes selected from transcriptome analysis (see Figure 4A: NME1-EV vs. Co-EV treatment) reveals that most of the pairwise comparisons are statistically significant. Figure S4: One-way ANOVA analysis of genes examined by qPCR (see Figure 4D: NME2-EV vs. Co-EV treatment) reveals that most of the pairwise comparisons are statistically significant. Figure S5: One-way ANOVA analysis of genes examined by qPCR (see Figure 4C: NME1-EV vs. Co-EV treatment) reveals that most of the pairwise comparisons are statistically significant.
