# Supplementary figures and images for: Extracellular Vesicle-Mediated Metastasis Suppressors NME1 and NME2 Modify Lipid Metabolism in Fibroblasts
Source: Cancers (Basel). 2022 Aug 13;14(16):3913. doi: 10.3390/cancers14163913 (PMC9406105; doi:10.3390/cancers14163913)

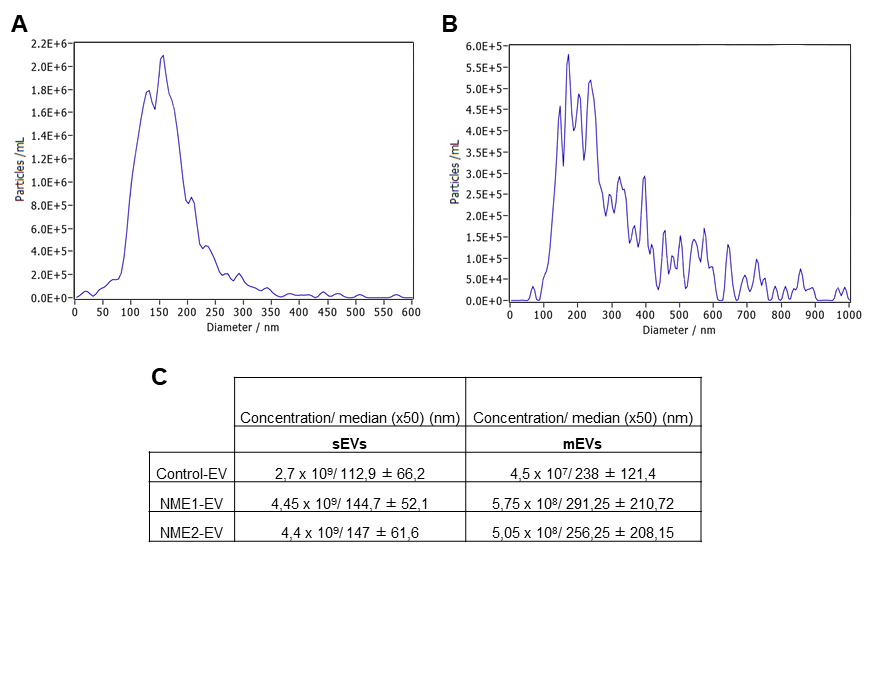

Supplement: Supplementary file 1 [file cancers-14-03913-s001.zip › Figure S1.tif]

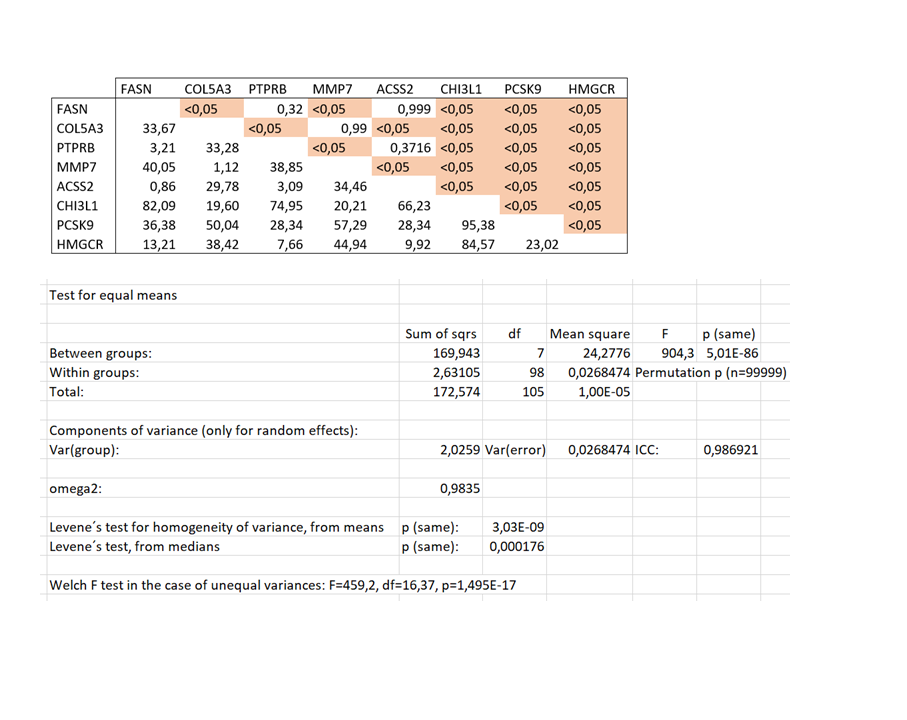

Supplement: Supplementary file 1 [file cancers-14-03913-s001.zip › Figure S2.tif]

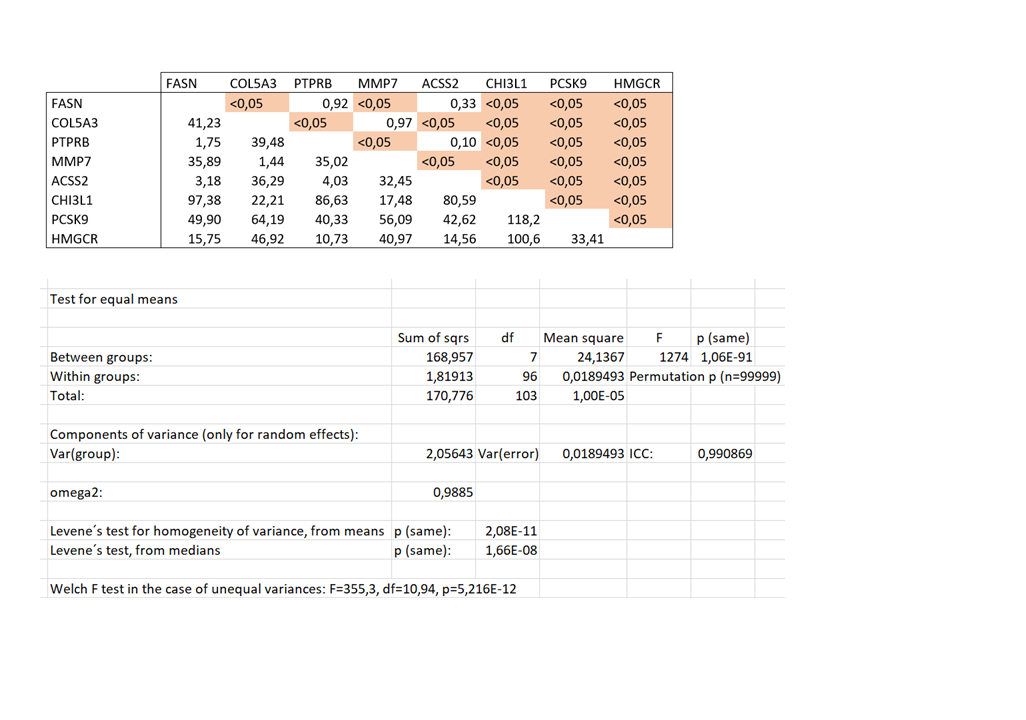

Supplement: Supplementary file 1 [file cancers-14-03913-s001.zip › Figure S3.tif]

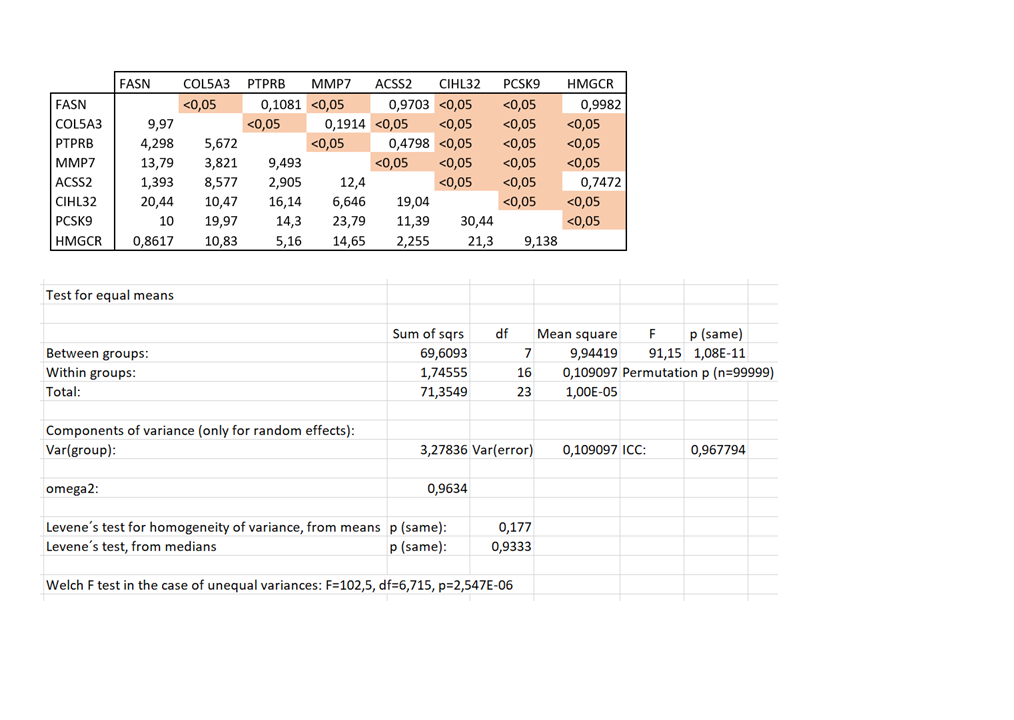

Supplement: Supplementary file 1 [file cancers-14-03913-s001.zip › Figure S4.tif]

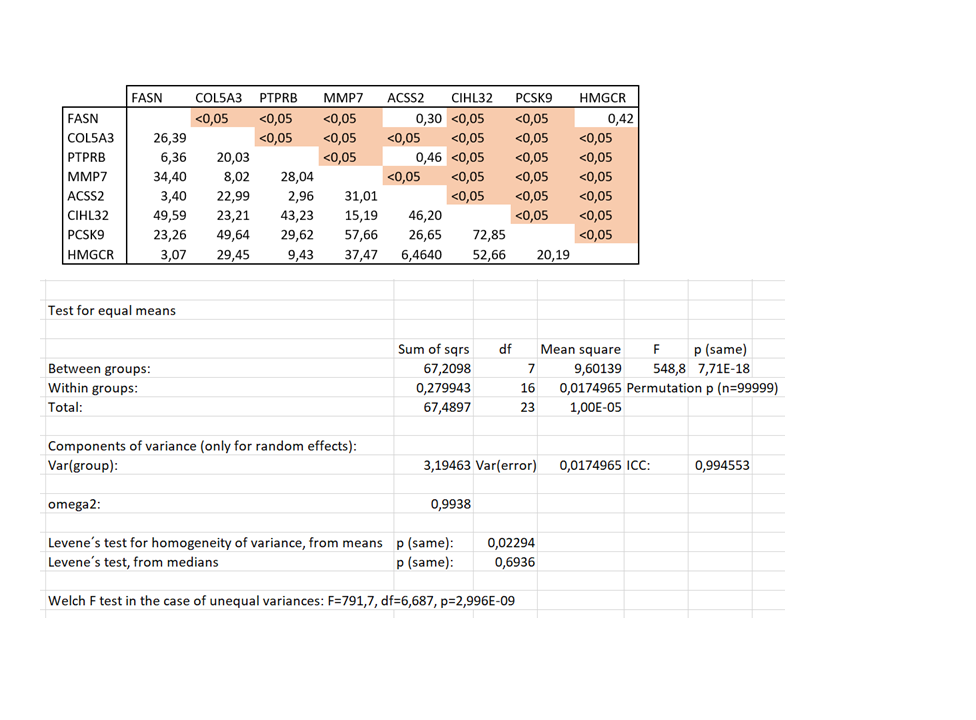

Supplement: Supplementary file 1 [file cancers-14-03913-s001.zip › Figure S5.tif]
